# Deep learning model for the automatic classification of COVID-19 pneumonia, non-COVID-19 pneumonia, and the healthy: a multi-center retrospective study

**DOI:** 10.1038/s41598-022-11990-3

**Published:** 2022-05-17

**Authors:** Mizuho Nishio, Daigo Kobayashi, Eiko Nishioka, Hidetoshi Matsuo, Yasuyo Urase, Koji Onoue, Reiichi Ishikura, Yuri Kitamura, Eiro Sakai, Masaru Tomita, Akihiro Hamanaka, Takamichi Murakami

**Affiliations:** 1 Department of Radiology, Kobe University Graduate School of Medicine, 7-5-2 Kusunoki-cho, Chuo-ku, Kobe, 650-0017 Japan; 2 Department of Radiology, Kobe City Medical Center General Hospital, 2-1-1 Minatojimaminamimachi, Chuo-ku, Kobe, 650-0047 Japan; 3 Department of Diagnostic Radiology, Kobe City Nishi-Kobe Medical Center, 5-7-1 Kojidai, Nishi-ku, Kobe, 651-2273 Japan; 4 Department of Radiology, Hyogo Prefectural Kakogawa Medical Center, 203 Kanno-cho kanno, Kakogawa, 675-8555 Japan; 5 Department of Radiology, Kita Harima Medical Center, 926-250 Ichiba-cho, Ono, 675-1392 Japan; 6 Department of Radiology, Hyogo Prefectural Awaji Medical Center, 1-1-137 Shioya, Sumoto, 656-0021 Japan

**Keywords:** Viral infection, Software

## Abstract

This retrospective study aimed to develop and validate a deep learning model for the classification of coronavirus disease-2019 (COVID-19) pneumonia, non-COVID-19 pneumonia, and the healthy using chest X-ray (CXR) images. One private and two public datasets of CXR images were included. The private dataset included CXR from six hospitals. A total of 14,258 and 11,253 CXR images were included in the 2 public datasets and 455 in the private dataset. A deep learning model based on EfficientNet with noisy student was constructed using the three datasets. The test set of 150 CXR images in the private dataset were evaluated by the deep learning model and six radiologists. Three-category classification accuracy and class-wise area under the curve (AUC) for each of the COVID-19 pneumonia, non-COVID-19 pneumonia, and healthy were calculated. Consensus of the six radiologists was used for calculating class-wise AUC. The three-category classification accuracy of our model was 0.8667, and those of the six radiologists ranged from 0.5667 to 0.7733. For our model and the consensus of the six radiologists, the class-wise AUC of the healthy, non-COVID-19 pneumonia, and COVID-19 pneumonia were 0.9912, 0.9492, and 0.9752 and 0.9656, 0.8654, and 0.8740, respectively. Difference of the class-wise AUC between our model and the consensus of the six radiologists was statistically significant for COVID-19 pneumonia (*p* value = 0.001334). Thus, an accurate model of deep learning for the three-category classification could be constructed; the diagnostic performance of our model was significantly better than that of the consensus interpretation by the six radiologists for COVID-19 pneumonia.

## Introduction

The novel coronavirus disease (COVID-19) outbreak is caused by a strain of coronavirus known as the severe acute respiratory syndrome coronavirus 2 that originated in Wuhan in the Hubei province in China at the end of 2019^1^. The World Health Organization declared COVID-19 as a pandemic on March 11, 2020, then it had spread across the world^2^. The website of the World Health Organization has listed the total number of reported patients with COVID-19 and the associated deaths. At the time of writing this paper, 163,869,893 patients and 3,398,302 deaths were reported on the website^3^.

COVID-19 is diagnosed using real-time polymerase chain reaction (RT-PCR) in many clinical situations. However, RT-PCR sensitivity is not very high in the detection of COVID-19; for example, one study reported that the sensitivity of RT-PCR (71%) was lower than that of chest computed tomography (98%)^4^. Owing to the low RT-PCR sensitivity, the effectiveness of chest X-Ray imaging (CXR) and computed tomography in the diagnosis of COVID-19 has been investigated^5^. The combination of CXR and artificial intelligence, such as deep learning (DL)^6^, has been extensively examined for automatic diagnosis of COVID-19^7–14^. Since CXR is widely available and its cost is relatively low, the combination of CXR and artificial intelligence could be employed for screening purposes of COVID-19 without the need for medical doctors.

Recent advances in DL have shown promising diagnostic performance for automatic classification of various diseases of the skin, retinal fundus, brain, and other organs^6,15–17^. DL-based automatic diagnosis is reportedly accurate, and performed well in the classification of COVID-19 pneumonia, non-COVID-19 pneumonia, and the healthy on CXR images^7–13^. Elgendi et al. compared the performance of 17 DL models with and without different geometric augmentations and examined the influence of data augmentation with respect to automatic classification of COVID-19 pneumonia. Their results demonstrated that the removal of the geometrical augmentation steps actually improved the performance of the DL models^13^. Monshi et al. optimized the data augmentation and the DL hyperparameters for classifying COVID-19 pneumonia. Their proposed CovidXrayNet based on EfficientNet-B0 achieved state-of-the-art accuracy^18^. Karakanis et al. proposed a new approach to classify COVID-19 pneumonia by exploiting a conditional generative adversarial network that generated synthetic images for augmenting the limited data amount. Their lightweight DL model (ResNet8-based) achieved competitive performance^19^. These technical advances of DL make the classification models of COVID-19 pneumonia more accurate and robust. However, the performance of DL models was mainly investigated using the public database of CXR, and the comparison of the diagnostic performance between DL models and radiologists was limited^14^.

Our study aimed to develop and validate a DL model for the automatic diagnosis of COVID-19 pneumonia, non-COVID-19 pneumonia, and the healthy using CXR images. In order to develop and validate our DL model, two public datasets and one private dataset of CXR images were implemented in the current study; CXR images of the private dataset were collected from six hospitals. To compare the diagnostic performance, both our DL model and six radiologists evaluated the CXR images of the private dataset. In addition, code-available DL models for diagnosing COVID-19 were also compared with our DL model. The major contributions of this study were as follows. (i) The two large public datasets of CXR images were constructed, which can be available online. (ii) Our DL model was validated with CXR images of our private dataset of clinical cases. (iii) The comparison of diagnostic performance was performed between our DL model and six radiologists.

## Methods

This retrospective study was approved by the institutional review boards of six hospitals (Kobe University Graduate School of Medicine, Kobe City Medical Center General Hospital, Kobe City Nishi-Kobe Medical Center, Hyogo Prefectural Kakogawa Medical Center, Kita Harima Medical Center, and Hyogo Prefectural Awaji Medical Center); the requirement for acquiring informed consent was waived owing to the retrospective nature of the stud. This study complied with the Declaration of Helsinki and Ethical Guidelines for Medical and Health Research Involving Human Subjects in Japan (https://www.mhlw.go.jp/file/06-Seisakujouhou-10600000-Daijinkanboukouseikagakuka/0000080278.pdf).

### Proposed DL model

**Figure 1:**
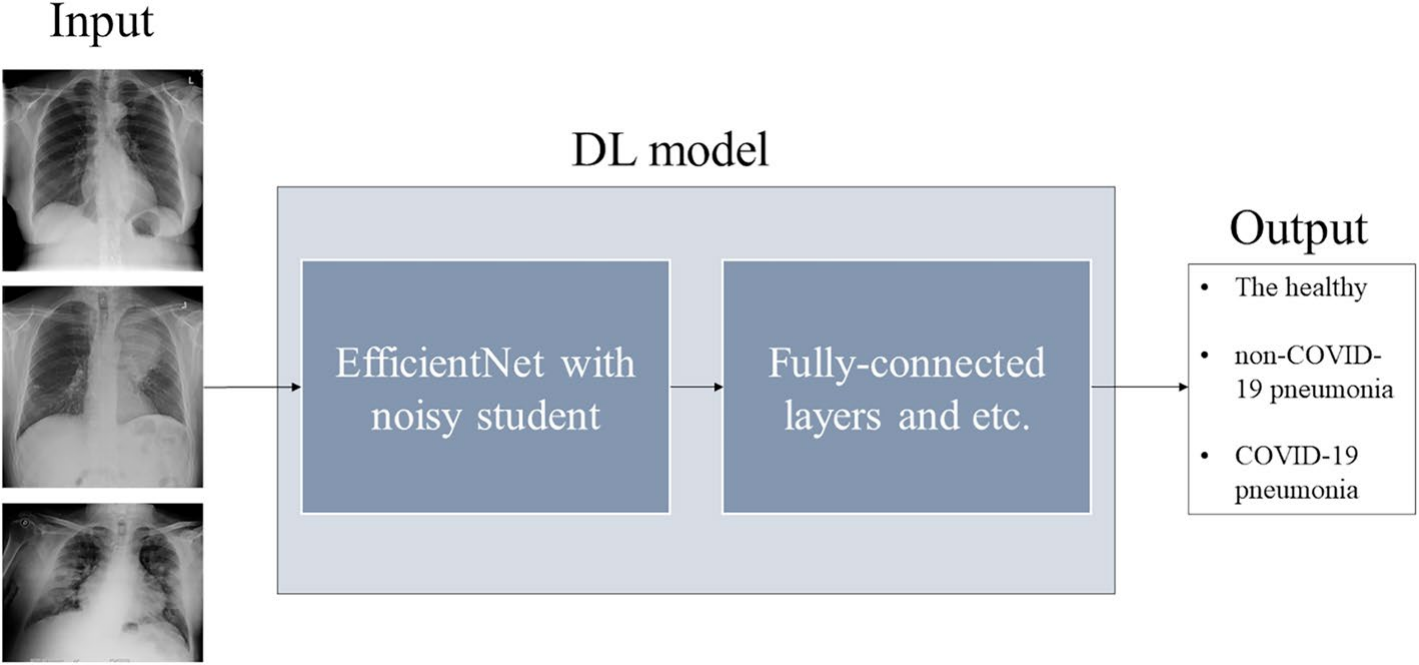
Our DL model. Abbreviation: DL, deep learning.

EfficientNet^20^ was used as our DL model. By use of the EfficientNet B5 pretrained with noisy student^21^, transfer learning was performed for the automatic classification of CXR images of COVID-19, non-COVID-19 pneumonia, and the healthy. The implementation of our DL model was based on the open-source software (https://github.com/jurader/covid19_xp) of a prior study^10^. While VGG16^22^ was used as the pretrained model in the prior study^10^, EfficientNet with noisy student was used in the current study. The outline of the DL model is shown in Fig. 1. The details of the DL model are described in the Supplementary information. Grad-CAM was used for visual explanation of the diagnosis by our DL model^23^.

### Datasets

CXR images with anterior–posterior or posterior-anterior views of two public datasets and one private dataset were implemented in the current study. One public dataset was the COVIDx dataset^12,24^. The other public dataset was constructed from two public datasets: the PadChest dataset^25,26^ and BIMCV-COVID19 + dataset^27,28^. Hereafter, we will refer to the second public dataset as COVID_BIMCV_. CXR images of the private dataset (COVID_private_) were retrospectively collected from the six hospitals. The details of the three obtained datasets are described in the Supplementary information.

Table 1 shows the total number of CXR images and the number of CXR images of COVID-19 pneumonia, non-COVID-19 pneumonia, and the healthy in the COVIDx, COVID_BIMCV_, and COVID_private_ datasets, respectively. The total number of CXR images was 14,258, 11,253, and 455 in the COVIDx, COVID_BIMCV_, and COVID_private_ datasets, respectively. The number of COVID-19 pneumonia cases were 617, 1475, and 177 in the COVIDx, COVID_BIMCV_, and COVID_private_ datasets, respectively.

**Table 1:** Numbers of CXR images in the COVIDx, COVID_BIMCV_, and COVID_private_ datasets.

Dataset	Total number of CXR images	Number of CXR images of the healthy	Number of CXR images of non-COVID-19 pneumonia	Number of CXR images of COVID-19 pneumonia
COVIDx	14,258	8066	5575	617
COVID_BIMCV_	11,253	8799	979	1475
COVID_private_	455	139	139	177

All cases of non-COVID-19 pneumonia are bacterial pneumonia in COVID_private_.

*CXR* chest X-Ray imaging; *COVIDx* public dataset used for COVID-Net; *COVID*_*BIMCV*_ public dataset obtained from the PadChest dataset and the BIMCV-COVID19 + dataset; *COVID*_*private*_ private dataset collected from six hospitals.

The patient characteristics of the COVID_private_ dataset are shown in Table 2. The number of CXR images of the healthy, non-COVID-19 pneumonia, and COVID-19 pneumonia in the COVID_private_ dataset was 139, 139, and 177, respectively. The COVID_private_ dataset included 198 males and 257 females, aged 61.0 ± 18.6 years. The examination date of CXR in the COVID_private_ dataset ranged from January 13th, 2015 to December 22th, 2020.

**Table 2:** Patients’ characteristics in the COVID_private_ dataset.

Hospital	Number of patients	Male	Female	Age (y) (mean ± standard deviation)
Hospital 1	6	4	2	68.0 ± 9.78
Hospital 2	20	15	5	61.7 ± 14.8
Hospital 3	7	5	2	73.1 ± 12.1
Hospital 4	173	104	69	58.3 ± 19.3
Hospital 5	186	99	87	61.2 ± 18.5
Hospital 6	63	30	33	65.3 ± 17.7
Total	455	198	257	61.0 ± 18.6

*COVID*_*private*_ private dataset collected from six hospitals.

### Dataset splitting and model training

Since the development set and test set were defined for the COVIDx dataset, they were used in the current study. A total of 100 and 50 CXR images were randomly selected as test sets for each of the COVID-19 pneumonia, non-COVID-19 pneumonia, and the healthy, in the COVID_BIMCV_ and COVID_private_ datasets, respectively. The other CXR images were used as development sets in the COVID_BIMCV_ and COVID_private_ datasets. Thus, the number of CXR images of the development set was 13,958, 10,953, and 305 in the COVIDx, COVID_BIMCV_, and COVID_private_ datasets, respectively. The test set size was 300 in the COVIDx and COVID_BIMCV_ datasets, and 150 in the COVID_private_ dataset.

**Figure 2:**
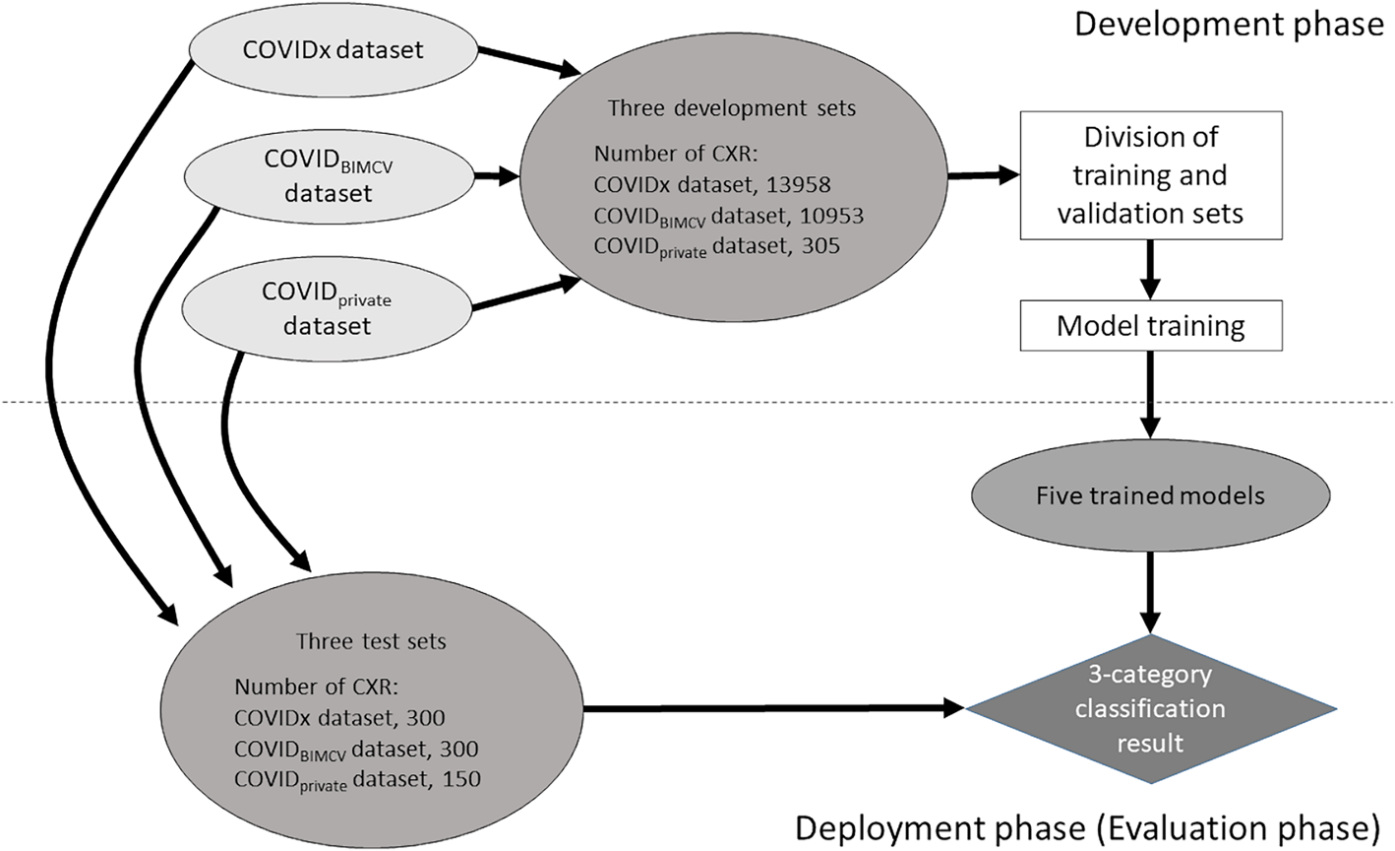
Schematic illustration of dataset splitting, model training, and prediction with our DL model. Abbreviations: COVIDx, Public dataset used for COVID-Net; COVIDBIMCV, Public dataset obtained from the PadChest dataset and the BIMCV-COVID19 + dataset; COVIDprivate, Private dataset collected from six hospitals.

The development set was further divided into a training and validation set for each dataset. The validation set size was 300 in the COVIDx and COVID_BIMCV_ datasets, and 90 in the COVID_private_ dataset. The combined training set was constructed from the training sets of the three datasets for training the DL model. For the development set, five different random divisions of training and validation sets were performed for each dataset. Based on the five random divisions, model training with transfer learning and performance validation were performed. Therefore, five different trained models were obtained. In order to predict the diagnosis from the CXR image of the test set, an ensemble of the five trained models was used. Schematic illustration of the dataset splitting, model training, and prediction using our DL model is shown in Fig. 2.

### Comparison with other DL models

Three code-available DL models were used for comparison. The first model was the COVID-Net model trained with the COVIDx dataset^12^. Its pretrained model is available at https://github.com/lindawangg/COVID-Net (COVIDNet-CXR4-A). The second model was the DL model of Sharma A et al.^11^, whose pretrained model is available at https://github.com/arunsharma8osdd/covidpred (Combined model 3 [101 epochs]). The final model was the DarkCovidNet^9^, which is available at https://github.com/muhammedtalo/COVID-19. Since the pretrained model of DarkCovidNet was unavailable, its model training was performed from scratch by the authors.

### Observer study by the radiologists

In order to compare our DL model with the radiologists’ diagnostic ability, an observer study was performed including six radiologists (experience of the six radiologists ranged from 10 months to 15 years). The radiologists visually evaluated the CXR images of the test set of the COVID_private_ dataset and determined the diagnosis for the three-category classification of COVID-19 pneumonia, non-COVID-19 pneumonia, and the healthy. With the exception of the CXR images, the radiologists were blinded to any clinical information of the test set of the COVID_private_ dataset. Since the combined training set used for our DL model was too large for the radiologists, the development set of the COVID_private_ dataset were provided for the radiologists’ training before the observer study. The training and interpretation time were not limited.

### Performance evaluation

For our DL model, performance evaluation was conducted using the classification metrics of the three-category classification (class-wise precision, recall, F1-score, and three-category classification accuracy) in the three test sets^29^. For radiologists and the code-available DL models, the same performance evaluation was conducted in the test set of the COVID_private_ dataset with 150 CXR images. In addition, the class-wise area under the curve (AUC) of the receiver operating characteristics (ROC) analysis was calculated for COVID-19 pneumonia, non-COVID-19 pneumonia, and the healthy^29^. For the ROC analysis of the radiologists, a consensus interpretation score for the six radiologists was determined by majority voting of the individual interpretations^14^; the score ranged from 0 to 6.

### Statistical analysis

The 95% confidence intervals (CI) of the classification metrics were calculated using 2000 bootstrap samples^14^. In addition, the class-wise AUC was compared using DeLong’s test between our DL model and the consensus interpretation of the radiologists. In order to control the family-wise error rate, Bonferroni correction was used; a *p* value less than 0.01666 was considered statistically significant. Statistical analyses were performed using scikit-learn package^30^ of Python and pROC package^31^ of R (version 4.0.4, https://www.r-project.org/).

## Results

Table 3 shows the results of the diagnostic performance of the four DL models, including our DL model, and the six radiologists in the test set of the COVID_private_ dataset. The three-category classification accuracy of our DL model was 0.8667 (130/150), and those of the six radiologists ranged from 0.5667 (85/150) to 0.7733 (116/150). The 95% CI of the three-category classification accuracies were 0.8067–0.9200 and 0.7067–0.8400 for our DL model and the radiologist with best accuracy (Radiologist 3), respectively. The three-category classification accuracy of our DL model was better than that of the six radiologists. For our DL model, the class-wise F1-scores of the healthy and COVID-19 pneumonia were higher than that of the non-COVID-19 pneumonia. This indicates that for our DL model, the diagnostic performance of the healthy and COVID-19 pneumonia was better than that of the non-COVID-19 pneumonia. On the other hand, for the six radiologists, the class-wise F1-scores of the healthy were higher than those of the COVID-19 pneumonia and non-COVID-19 pneumonia; hence, the diagnostic performance of the healthy was higher than that for COVID-19 and non-COVID-19 pneumonia. The three-category classification accuracies of the three code-available DL models were 0.6467 (97/150), 0.4267 (64/150), and 0.4000 (60/150), and COVID-Net^12^ achieved the highest accuracy in the three-category classification among the three code-available DL models. Although the three-category classification accuracy of COVID-Net (0.6467) was comparable to those of the six radiologists, those of the other code-available DL models (0.4267 and 0.4000) were worse than those of the six radiologists. The class-wise F1-scores of the three code-available DL models for COVID-19 pneumonia were 0.3636, 0.5684, and 0.4160, and the DL model of Sharma et al.^11^ achieved the highest class-wise F1-score for COVID-19 pneumonia among them; the class-wise F1-score of the DL model of Sharma et al. (0.5684) was higher than those of two radiologists (Radiologist 5 and Radiologist 6). However, the class-wise F1-score of the DL model of Sharma et al. for the healthy was 0.0000. Table S1 of the Supplementary information shows the results of the diagnostic performance in our DL model in the test sets of the COVIDx and COVID_BIMCV_ datasets.

**Table 3:** Class-wise precision, recall, F1-score, and three-category classification accuracy of four DL models and six radiologists in the COVID_private_ dataset.

Model or Radiologist	The healthy			Non-COVID-19 pneumonia			COVID-19 pneumonia			
	Precision	Recall	F1-score	Precision	Recall	F1-score	Precision	Recall	F1-score	Accuracy*
Our DL model	0.8475, 0.7458, 0.9348	1.0000, 1.0000, 1.0000	0.9174, 0.8544, 0.9663	0.8974, 0.7949, 0.9767	0.7000, 0.5652, 0.8302	0.7865, 0.6829, 0.8736	0.8654, 0.7609, 0.9512	0.9000, 0.8095, 0.9783	0.8824, 0.8049, 0.9412	0.8667, 0.8067, 0.9200
COVID-Net	0.6173, 0.5067, 0.7229	1.0000, 1.0000, 1.0000	0.7634, 0.6726, 0.8392	0.6604, 0.5254, 0.7827	0.7000, 0.5714, 0.8182	0.6796, 0.5656, 0.7708	0.7500, 0.5000, 0.9412	0.2400, 0.1250, 0.3636	0.3636, 0.2089, 0.5079	0.6467, 0.5667, 0.7200
Sharma et al	0.0000, 0.0000, 0.0000	0.0000, 0.0000, 0.0000	0.0000, 0.0000, 0.0000	0.3627, 0.2687, 0.4592	0.7400, 0.6121, 0.8605	0.4868, 0.3803, 0.5806	0.6000, 0.4524, 0.7500	0.5400, 0.3958, 0.6793	0.5684, 0.4337, 0.6813	0.4267, 0.3400, 0.5067
DarkCovidNet	0.2500, 0.0000, 1.0000	0.0200, 0.0000, 0.0638	0.0370, 0.0000, 0.1132	0.4648, 0.3478, 0.5882	0.6600, 0.5227, 0.7869	0.5455, 0.4301, 0.6462	0.3467, 0.2429, 0.4588	0.5200, 0.3799, 0.6591	0.4160, 0.3051, 0.5206	0.4000, 0.3267, 0.4800
Radiologist1	0.8039, 0.6862, 0.9038	0.8200, 0.7111, 0.9167	0.8119, 0.7209, 0.8837	0.6327, 0.4902, 0.7619	0.6200, 0.4878, 0.7547	0.6263, 0.5055, 0.7333	0.6400, 0.5088, 0.7727	0.6400, 0.5000, 0.7647	0.6400, 0.5238, 0.7358	0.6933, 0.6200, 0.7600
Radiologist2	0.8333, 0.7222, 0.9318	0.8000, 0.6779, 0.9038	0.8163, 0.7209, 0.8932	0.7000, 0.5714, 0.8197	0.7000, 0.5745, 0.8182	0.7000, 0.5895, 0.7959	0.7115, 0.5818, 0.8302	0.7400, 0.6111, 0.8519	0.7255, 0.6200, 0.8148	0.7467, 0.6800, 0.8133
Radiologist3	0.8600, 0.7547, 0.9512	0.8600, 0.7556, 0.9500	0.8600, 0.7755, 0.9250	0.7200, 0.5957, 0.8400	0.7200, 0.5882, 0.8409	0.7200, 0.6118, 0.8142	0.7400, 0.6154, 0.8667	0.7400, 0.6122, 0.8537	0.7400, 0.6316, 0.8367	0.7733, 0.7067, 0.8400
Radiologist4	0.6154, 0.5051, 0.7215	0.9600, 0.8965, 1.0000	0.7500, 0.6560, 0.8244	0.8276, 0.6786, 0.9615	0.4800, 0.3404, 0.6200	0.6076, 0.4706, 0.7246	0.6279, 0.4736, 0.7778	0.5400, 0.3921, 0.6724	0.5806, 0.4444, 0.6903	0.6600, 0.5865, 0.7333
Radiologist5	0.7358, 0.6122, 0.8511	0.7800, 0.6596, 0.8913	0.7573, 0.6531, 0.8432	0.5417, 0.4000, 0.6793	0.5200, 0.3846, 0.6563	0.5306, 0.4051, 0.6400	0.5102, 0.3725, 0.6471	0.5000, 0.3673, 0.6316	0.5051, 0.3789, 0.6154	0.6000, 0.5267, 0.6800
Radiologist6	0.5385, 0.4375, 0.6429	0.9800, 0.9362, 1.0000	0.6950, 0.6031, 0.7792	0.6667, 0.4783, 0.8519	0.3600, 0.2249, 0.5000	0.4675, 0.3158, 0.6001	0.5625, 0.3793, 0.7419	0.3600, 0.2222, 0.4894	0.4390, 0.2899, 0.5618	0.5667, 0.4867, 0.6467

Each cell includes classification metric and its 95% CI (lower and upper bounds of CI). * indicates 3-category classification accuracy. The experience of the six radiologists were 10 months, and 4, 7, 10, 10, and 15 years. The underlined values represent the best values for each column.

*DL* deep learning; *CI* confidence interval; *COVID*_*private*_ private dataset collected from six hospitals.

**Figure 3:**
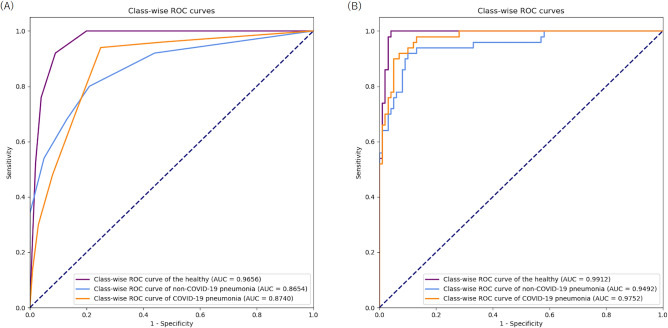
Class-wise ROC curves in COVID_private_ dataset. Note: (**A**) consensus of radiologists and (**B**) our DL model. Abbreviation: DL, deep learning; COVID_private_, private dataset collected from six hospitals; AUC, area under the curve; ROC, receiver operating characteristics.

Table 4 shows the results of class-wise AUC and its 95% CI of our DL model in the test sets of the COVIDx, COVID_BIMCV_, and COVID_private_ datasets. Table 4 also shows the results of the consensus of the six radiologists in the test set of the COVID_private_ dataset. Figure 3 shows the class-wise ROC curves of our DL model and consensus of the six radiologists in the test set of the COVID_private_ dataset. The class-wise AUC and its 95% CI of our DL model were as follows: 0.9914 and 0.9837–0.9990 for the healthy, 0.9772 and 0.9601–0.9942 for non-COVID-19 pneumonia, and 0.9934 and 0.9871–0.9996 for COVID-19 pneumonia. The class-wise AUC and its 95% CI of consensus of the six radiologists were as follows: 0.9656 and 0.9401–0.9911 for the healthy, 0.8654 and 0.8022–0.9286 for non-COVID-19 pneumonia, and 0.8740 and 0.8164–0.9316 for COVID-19 pneumonia. The difference of the class-wise AUC between our DL model and consensus of the six radiologists was statistically significant for COVID-19 pneumonia (*p* value = 0.001334). The differences were not statistically significant for the healthy and non-COVID-19 pneumonia (*p* values = 0.07252 and 0.02617, respectively). Table S2 of the Supplementary information presents the confusion matrix of the three-category classification for our DL model in the test set of the COVID_private_ dataset. Table S3 of the Supplementary information shows the class-wise AUC and its 95% CI for our DL model when changing the data splitting between the test and development sets. Figures S1 and S2 of the Supplementary information show the class-wise ROC curves of our DL model in the test sets of the COVIDx and COVID_BIMCV_ datasets, respectively.

**Table 4:** Class-wise AUC and its 95% CI of our DL model and consensus of six radiologists.

Model or Radiologist	Dataset	The healthy		Non-COVID-19 pneumonia		COVID-19 pneumonia	
		AUC	95% CI	AUC	95% CI	AUC	95% CI
Our DL model	COVIDx	0.9914	0.9837, 0.9990	0.9772	0.9601, 0.9942	0.9934	0.9871, 0.9996
Our DL model	COVID_BIMCV_	0.9712	0.9548, 0.9877	0.9568	0.9355, 0.9781	0.9856	0.9702, 1
Our DL model	COVID_private_	0.9912	0.9801, 1.0000	0.9492	0.9118, 0.9866	0.9752	0.9555, 0.9949
COVID-Net	COVID_private_	0.8917	0.8405, 0.9429	0.8500	0.7909, 0.9091	0.7167	0.6347, 0.7987
Sharma et al	COVID_private_	0.6074	0.5111, 0.7037	0.5017	0.4089, 0.5945	0.7564	0.6768, 0.8360
DarkCovidNet	COVID_private_	0.4315	0.3350, 0.5280	0.7226	0.6420, 0.8032	0.5589	0.4630, 0.6548
Consensus of radiologists	COVID_private_	0.9656	0.9401, 0.9911	0.8654	0.8022, 0.9286	0.8740	0.8164, 0.9316

*DL* deep learning; *CI* confidence interval; *AUC* area under the curve; *COVIDx* public dataset used for COVID-Net; *COVID*_*BIMCV*_ public dataset obtained from the PadChest dataset and the BIMCV-COVID19 + dataset; *COVID*_*private*_ private dataset collected from six hospitals.

**Figure 4:**
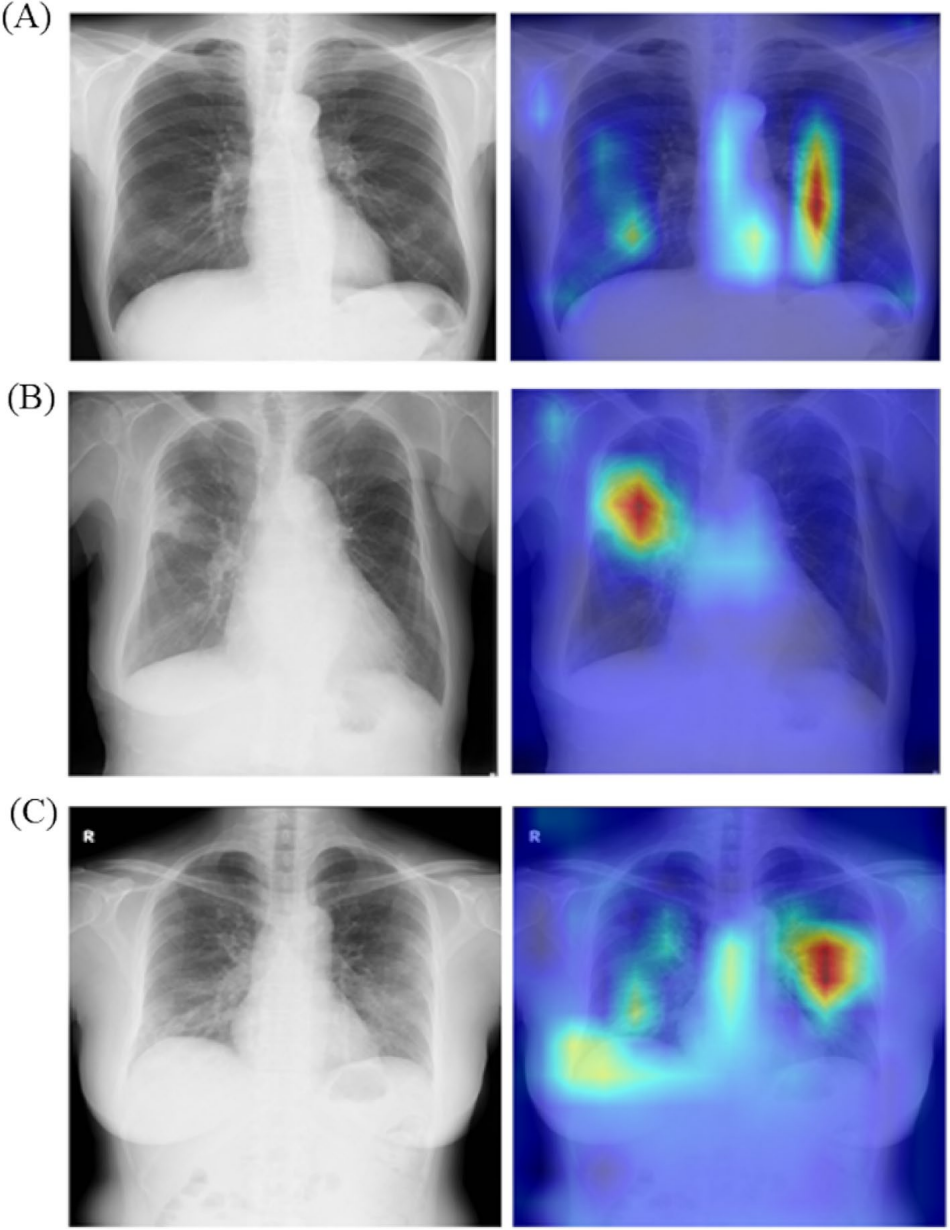
Results of Grad-CAM for our DL model. Note: (**A**) the healthy, (**B**) non-COVID-19 pneumonia, (**C**) COVID-19 pneumonia. Each image part consists of CXR image and result of Grad-CAM. One trained model of our DL model was used for Grad-CAM. Abbreviation: DL, deep learning; CXR, chest X-Ray imaging.

Figure 4 shows the CXR images and the results of Grad-CAM for the healthy, non-COVID-19 pneumonia, and COVID-19 pneumonia. The result of Grad-CAM of Fig. 4A illustrates that our DL model focused on the non-specific areas for diagnosis of the healthy. Figure 4B shows that our DL model focused on the infiltration shadow of the right lung field for diagnosis of non-COVID-19 pneumonia. Figure 4C shows that our DL model focused on the ground glass shadow of the peripheral area of both the lung fields for the diagnosis of COVID-19 pneumonia.

## Discussion

The results of this study indicate that it is possible to construct an accurate DL model using the two public datasets (COVIDx and COVID_BIMCV_) and one private dataset (COVID_private_). Our deep learning model based on EfficientNet with noisy student could achieve an accurate diagnosis of COVID-19 pneumonia, non-COVID-19 pneumonia, and the healthy. The three-category classification accuracy of our model was 0.8667, and those of the six radiologists ranged from 0.5667 to 0.7733. Difference of class-wise AUC between our model and the consensus of the six radiologists was statistically significant for COVID-19 pneumonia (*p* value = 0.001334).

Using the two public datasets and one private dataset, our DL model could achieve a higher diagnostic performance than the three code-available DL models and the six radiologists. Especially, for COVID-19 pneumonia, the class-wise AUC of our DL model was significantly higher than that of the consensus of the six radiologists. In DL, a large number of datasets is necessary for accurate classification. While COVID-Net used more than 10,000 CXR images to develop and evaluate its model^12^, we used more than 20,000 CXR images for our DL model. We believe that the dataset size was a major factor in the diagnostic performance of our DL model. Another reason for the superiority of our DL model could be attributed to the use of a pretrained model constructed using noisy student^21^. Noisy student is a relatively new method for increasing the robustness of the DL model; the pretrained model of EfficientNet^20^ with noisy student could be useful in improving our DL model.

The results of the three code-available DL models demonstrate that their classification metrics are not satisfactory. Although the three-category classification accuracy of COVID-Net was the highest in the three DL models, the F1-score of COVID-Net was the worst for COVID-19 pneumonia. In the other two models, the three-category classification accuracy was lower than those of the six radiologists. Many studies have used DL models for automatic classification of COVID-19 pneumonia, non-COVID-19 pneumonia, and the healthy using CXR images^7–14,18,19^. Table 5 summarizes these previous studies. While most of them were developed and validated using CXR images of public datasets, they were not validated with those of clinical cases. Our results indicate that most of the DL models of COVID-19 pneumonia in previously published papers may not be useful in clinical situations.

**Table 5:** Summary of COVID-19 DL models on CXR images.

Authors	Classification	Dataset	Number of COVID-19 images	Performance	Comparison with radiologists
Shorfuzzaman et al.^7^	Multi-class, Binary	Public	230	Accuracy = 95.6% (multi-class)	No
Ozturk et al.^9^	Multi-class, Binary	Public	125	Accuracy = 87.02% (multi-class)	No
Nishio et al.^10^	Multi-class	Public	215	Accuracy = 83.6%	No
Sharma et al.^11^	Multi-class	Public	51 (original) 75 (dataset-II)	COVID-19 Sensitivity = 100% COVID-19 Sensitivity = 66.67	No
Wang et al.^12^	Multi-class	Public	358 (original COVIDx)	Accuracy = 93.3%	No
Elgendi et al.^13^	Multi-class	Public, Private	50 (Dataset 1) 198 (Dataset 2) 248 (Dataset 3) 58 (Dataset 4)	MCC = 0.51	No
Wehbe et al.^14^	Binary	Private	4253	Accuracy = 82%	Yes
Monshi et al.^18^	Multi-class	Public	320 (COVIDcxr) NA (COVIDx ver. 3)	Accuracy = 95.82%	No
Karakanis et al.^19^	Multi-class, Binary	Public	145	Accuracy = 98.3%	No
Ours	Multi-class	Public, Private	617 (COVIDx ver. 5) 1475 (COVID_BIMCV_) 177 (COVID_private_)	Accuracy = 86.67%	Yes

Definition of accuracy in multi-class classification may be different between these studies.

*CXR* chest X-Ray imaging; *DL* deep learning; *NA* not available; *MCC* Matthews correlation coefficient; *COVIDx* public dataset used for COVID-Net.

The three-category classification accuracy of the six radiologists ranged from 0.5667 to 0.7733. There was large variability in the diagnostic performance of the radiologists in the classification of COVID-19 pneumonia, non-COVID-19 pneumonia, and the healthy using CXR images. Inversely, this indicates that the radiologists’ diagnostic performance could be improved using our DL model. The effectiveness of our DL model for computer-aided diagnosis system should be evaluated in future studies.

There are certain limitations to our study. First, although our DL model was developed and validated using two public datasets and one private dataset, it was not evaluated using external validation. Clinical usefulness of our DL model should be further evaluated by external validation^32^. Second, our DL model focused on the three-category classification of COVID-19 pneumonia, non-COVID-19 pneumonia, and the healthy. The DL model ignored lung cancer and other diseases, which are considered important for detection on CXR images. This three-category classification may be considered unnatural from a clinical viewpoint. However, we speculate that this was justified owing to the higher priority of the three-category classification in the COVID-19 pandemic. Third, our observer study was conducted on the CXR image obtained from relatively large-sized hospitals. However, since CXR can be performed in various hospitals and clinics, further studies are warranted to determine whether our DL model is effective in small hospitals and clinics. Thus, the outputs of our DL model should be adjusted based on the circumstances in which our DL model is used. Fourth, we focused on the automatic classification of COVID-19 pneumonia, non-COVID-19 pneumonia, and the healthy using CXR images and the diagnostic performance of radiologists with our DL model was not evaluated. Thus, we did not evaluate the usefulness of our DL model as a computer-aided system. If radiologists doubt the results of our DL model, the diagnostic performance of radiologists may not be improved using our DL model. Therefore, in the future, it is crucial to build trust between the radiologists and the DL model for its implementation in clinical practice^33^. Fifth, although the results of Grad-CAM (for example, Fig. 4) could be useful to radiologists for comprehending the classification results of our DL model, the effectiveness of the results of Grad-CAM was not validated in the current study.

In conclusion, it is feasible to create an accurate model of DL for three-category classification of COVID-19 pneumonia, non-COVID-19 pneumonia, and the healthy. The diagnostic performance of our model was significantly better than that of the consensus interpretation by the six radiologists for COVID-19 pneumonia.

## Supplementary Information

Supplementary Information.

## Data Availability

The private dataset cannot be disclosed because of privacy protection and regulation. Source code of our DL model and the two public datasets are available from the following URL: https://github.com/jurader/covid19_xp_efficientnet.

## Abbreviations

COVID-19: Novel coronavirus disease
RT-PCR: Real-time polymerase chain reaction
CXR: Chest X-ray imaging
DL: Deep learning
COVIDx: Public dataset used for COVID-Net
COVID_BIMCV_: Public dataset obtained from the PadChest dataset and the BIMCV-COVID19 + dataset
COVID_private_: Private dataset collected from six hospitals
AUC: Area under the curve
ROC: Receiver operating characteristics
CI: Confidence interval
