# Supplementary material for: Deep learning model for the automatic classification of COVID-19 pneumonia, non-COVID-19 pneumonia, and the healthy: a multi-center retrospective study
Source: Sci Rep. 2022 May 17;12:8214. doi: 10.1038/s41598-022-11990-3 (PMC9113076; doi:10.1038/s41598-022-11990-3)
Supplement: Supplementary file 1 — Supplementary Information. [file 41598_2022_11990_MOESM1_ESM.docx]

**Supplemental Material**

**Detail of proposed DL model**

Our DL model was based on open-source software of a prior study (1). The difference between DL model of ours and that of the prior study was minor. The layers of EfficientNet were sorted in the order of image processing, and trainable parameters was frozen for the first 30% layers of EfficientNet. Next, as in the prior study, the global averaging pooling layer, fully-connected layer, and dropout layer were added, after the convolution layers of EfficientNet. For the 3-category classification, the final fully-connected layer was added after the dropout layer. Activation functions of the first and second fully-connected layer were rectified linear unit and softmax, respectively.

Network architecture and hyperparameters of our DL model were as follows.

- EfficientNet B5 pretrained with noisy student was used.
- The probability of the dropout layer was 0.3.
- The number of outputs in the first fully-connected layer was 416.
- ADAM with learning rate of 6.0 × 10^-5^ was used as the optimizer.
- Cross entropy loss was used.
- The input image size was 456 × 456 pixels.
- The batch size was 9.
- The number of training epochs was set to 50.
- Number of CXR images used for one-epoch training was 50000.
- For each training epoch, the ratio of CXR images used for training our model was set to 1:3 between the two public datasets and COVID_private_ dataset.
- Early stopping was enabled using validation loss, and the patience of early stopping was set to 8. Validation loss was calculated using validation set of the COVID_private_ dataset.

To prevent overfitting in the model training, the combination of two types of data augmentation methods (conventional method, mixup [2]) was used.

The conventional data augmentation method included the following image processing.

- ±2° rotation,
- ±2% x-axis shift,
- ±2% y-axis shift,
- 98%–102% scaling.

The parameter of mixup was set to 0.4.

The training of our DL model was performed using a workstation with a discrete GPU (Nvidia RTX 2080 Ti, RAM 11 GB). Python (version 3.7, http://www.python.org/) was used as the programing language, and Keras (version 2.4.3, http://keras.io/) and TensorFlow (version 2.4.0, http://tensorflow.org/) were used as deep learning frameworks.

**Detail of datasets**

CXR images with anterior-posterior or posterior-anterior view of two public datasets and one private dataset were used for the current study. One public dataset was the COVIDx dataset (3). The other public dataset was constructed from two public datasets: PadChest dataset (4) and BIMCV-COVID19+ dataset (5). CXR images of the private dataset were retrospectively collected from six hospitals.

The COVIDx dataset was a large open dataset in terms of the number of COVID-19 positive patient cases (3). The COVIDx dataset was used to train and evaluate COVID-Net model (3). It consists of COVID-19 pneumonia, non-COVID-19 pneumonia, and the healthy. The COVIDx dataset was constructed from five different publicly available data repositories. The way to construct the COVIDx dataset is disclosed as open-source software (https://github.com/lindawangg/COVID-Net). While there are several versions of the COVIDx dataset, version 5 was utilized in the current study.

The PadChest dataset is a labeled large-scale dataset of CXR images. The PadChest dataset includes more than 160,000 images obtained from 67,000 patients that were interpreted and reported by radiologists from 2009 to 2017. 19 differential diagnoses were labeled in the PadChest dataset. Because 27% were manually annotated by trained physicians in the PadChest dataset, only the manually annotated data were selected in the current study. In addition, CXR images of non-COVID-19 pneumonia and the healthy were selected from the PadChest dataset for the current study. BIMCV-COVID19+ dataset is a large dataset with CXR and computed tomography images of COVID-19 patients. Dataset size was increased in the BIMCV-COVID19+ dataset after we obtained the images of BIMCV-COVID19+ dataset. CXR images of COVID-19 pneumonia were selected from the BIMCV-COVID19+ dataset in the current study. By combining CXR images of non-COVID-19 pneumonia and the healthy from the PadChest dataset and those of COVID-19 pneumonia from the BIMCV-COVID19+ dataset, the second public dataset was constructed.

CXR images of the private dataset (COVIDprivate) were collected from the six hospitals. The inclusion criteria of COVIDprivate were as follows; (i) for COVID-19 pneumonia, RT-PCR was positive; (ii) for COVID-19 pneumonia, the date difference between RT-PCR and CXR was less than 7 days. (iii) for non-COVID-19 pneumonia, non-COVID-19 pneumonia was confirmed clinically (for example, positive results of sputum culture and/or effectiveness of antibiotics were confirmed in bacterial pneumonia) (iv) for non-COVID-19 pneumonia, the date difference between clinical diagnosis of non-COVID-19 pneumonia and CXR was less than 7 days; (v) for the healthy, no abnormal findings were confirmed on CXR images. In addition, subsequent CXR and/or CT also showed no abnormal findings; (vi) for non-COVID-19 pneumonia and the healthy, the date of CXR images was before 2019/08/31; this criterion was applied for excluding potential false negatives of RT-PCR; (vii) for COVID-19 pneumonia and non-COVID-19 pneumonia, CXR was performed before or immediately after treatment; (viii) view of CXR was anterior-posterior or posterior-anterior. The exclusion criterion was (i) patient age <20.

**References**

1. Nishio M, Noguchi S, Matsuo H, Murakami T. Automatic classification between COVID-19 pneumonia, non-COVID-19 pneumonia, and the healthy on chest X-ray image: combination of data augmentation methods. Sci Rep. Nature Research; 2020;10(1):1–6. doi: 10.1038/s41598-020-74539-2.

2. Zhang H, Cisse M, Dauphin YN, Lopez-Paz D. mixup: Beyond Empirical Risk Minimization. Polit Africaine. 2017;1–13. http://arxiv.org/abs/1710.09412. Accessed May 27, 2020.

3. Wang L, Lin ZQ, Wong A. COVID-Net: a tailored deep convolutional neural network design for detection of COVID-19 cases from chest X-ray images. Sci Rep. Nature Research; 2020;10(1):1–12. doi: 10.1038/s41598-020-76550-z.

4. Bustos A, Pertusa A, Salinas JM, de la Iglesia-Vayá M. PadChest: A large chest x-ray image dataset with multi-label annotated reports. Med Image Anal. Elsevier B.V.; 2020;66:101797. doi: 10.1016/j.media.2020.101797.

5. Vayá M de la I, Saborit JM, Montell JA, et al. BIMCV COVID-19+: a large annotated dataset of RX and CT images from COVID-19 patients. arXiv. arXiv; 2020; http://arxiv.org/abs/2006.01174. Accessed April 26, 2021.

**Figure S1**

Class-wise ROC curves of our DL model in COVIDx dataset.


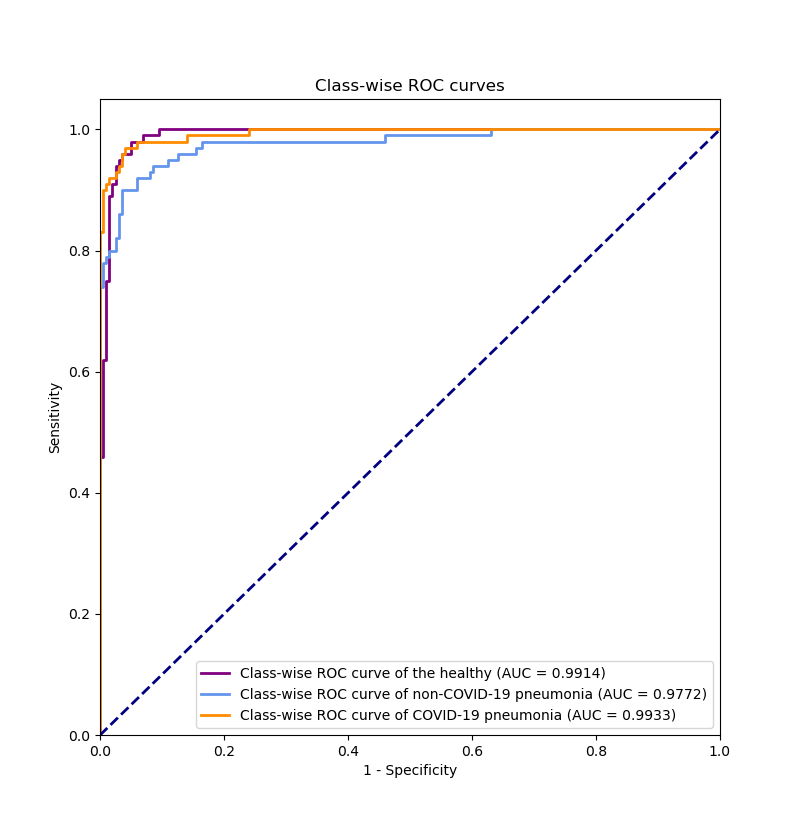


**Figure S2**

Class-wise ROC curves of our DL model in COVID_BIMCV_ dataset.


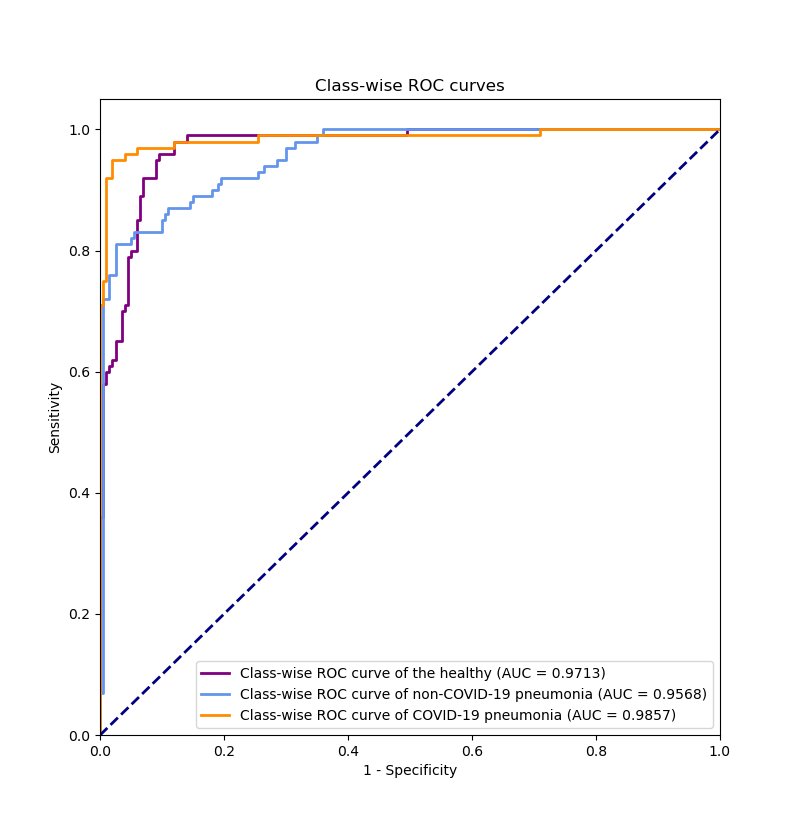


**Table S1**

Class-wise precision, recall, F1-score, and 3-category classification accuracy of our DL model in COVIDx and COVID_BIMCV_ datasets.

|  | the healthy | | | non-COVID-19 pneumonia | | | COVID-19 pneumonia | | |  |
| --- | --- | --- | --- | --- | --- | --- | --- | --- | --- | --- |
| Dataset | precision | recall | F1-score | precision | recall | F1-score | precision | recall | F1-score | Accuracy^*^ |
| COVIDx | 0.8909, 0.8276, 0.9487 | 0.9800, 0.9500, 1.0000 | 0.9333, 0.8947, 0.9668 | 0.8824, 0.8172, 0.9417 | 0.9000, 0.8364, 0.9556 | 0.8911, 0.8426, 0.9321 | 0.9886, 0.9634, 1.0000 | 0.8700, 0.8021, 0.9320 | 0.9255, 0.8823, 0.9604 | 0.9167, 0.8833, 0.9467 |
| COVID_BIMCV_ | 0.7226, 0.6467, 0.7937 | 0.9900, 0.9681, 1.0000 | 0.8354, 0.7809, 0.8814 | 0.9844, 0.9454, 1.0000 | 0.6300, 0.5306, 0.7257 | 0.7683, 0.6883, 0.8364 | 0.9596, 0.9135, 0.9907 | 0.9500, 0.9029, 0.9893 | 0.9548, 0.9231, 0.9811 | 0.8567, 0.8167, 0.8933 |

Note: Each cell includes classification metric and its 95% CI (lower and upper bounds of CI). * indicates 3-category classification accuracy.

Abbreviation: DL, convolutional neural network; CI, confidence interval; COVIDx, public dataset used for COVID-Net; COVID_BIMCV_, public dataset obtained from the PadChest dataset and the BIMCV-COVID19+ dataset.

**Table S2**

Confusion matrix of three-category classification of our DL model in test set of COVID_private_ dataset.

|  |  | Prediction by our DL model | | |
| --- | --- | --- | --- | --- |
|  |  | the healthy | non-COVID-19 pneumonia | COVID-19 pneumonia |
| Ground truth | the healthy | 50 | 0 | 0 |
|  | non-COVID-19 pneumonia | 8 | 35 | 7 |
|  | COVID-19 pneumonia | 1 | 4 | 45 |

Table S3

Class-wise AUC and its 95% CI of our DL model when changing the data splitting randomly between test sets and development sets.

|  |  | the healthy | | non-COVID-19 pneumonia | | COVID-19 pneumonia | |
| --- | --- | --- | --- | --- | --- | --- | --- |
| Model | Dataset | AUC | 95% CI | AUC | 95% CI | AUC | 95% CI |
| Our DL model | COVIDx | 1.000 | 1.000, 1.000 | 0.9984 | 0.9956, 1.000 | 1.000 | 1.000, 1.000 |
| Our DL model | COVID_BIMCV_ | 0.9560 | 0.9353, 0.9768 | 0.9192 | 0.8859, 0.9525 | 0.9978 | 0.9955, 1.000 |
| Our DL model | COVID_private_ | 0.9700 | 0.9445, 0.9955 | 0.9158 | 0.8672, 0.9644 | 0.9672 | 0.9448, 0.9896 |

Abbreviation: DL, deep learning; CI, confidence interval; AUC, area under the curve; COVIDx, public dataset used for COVID-Net; COVID_BIMCV_, public dataset obtained from the PadChest dataset and the BIMCV-COVID19+ dataset; COVID_private_, private dataset collected from six hospitals.

**Table S4**

Confusion matrix of three-category classification of a radiologist in test set of COVID_private_ dataset.

|  |  | Prediction by radiologist | | |
| --- | --- | --- | --- | --- |
|  |  | the healthy | non-COVID-19 pneumonia | COVID-19 pneumonia |
| Ground truth | the healthy | 41 | 7 | 2 |
|  | non-COVID-19 pneumonia | 3 | 31 | 16 |
|  | COVID-19 pneumonia | 7 | 11 | 32 |

Note: The accuracy of this radiologist is 69.3%.
